# Mental health among Iranian combat veterans with ankle-foot neuromusculoskeletal injuries

**DOI:** 10.1186/s40779-017-0138-1

**Published:** 2017-09-26

**Authors:** Arsia Taghva, Mostafa Allami, Kamyab Alizadeh, Anahita Zandi, Elahe Faraji, Zohreh Ganjparvar

**Affiliations:** 1 AJA University of Medical Sciences, Disaster and Military Psychiatry Research Center, Tehran, Iran; 2 Janbazan Medical and Engineering Research Center, Tehran, Iran; 3 Nursing and Midwifery Department, Torbat Heydarieh University of Medical Sciences, Torbat, Iran

**Keywords:** Veterans’ health, Ankle, Foot, Mental health

## Abstract

**Background:**

Veterans with purely physical disorders, such as ankle-foot neuromusculoskeletal disorders, are often neglected in psychological assessments because mental health evaluations are usually focused on those with a psychological disturbance or with a high percentage of injury. The purpose of this study was to evaluate the psychological condition of veterans with ankle-foot neuromusculoskeletal disorders.

**Methods:**

A cross-sectional study was performed between 2014 and 2016 on veterans with war-related ankle-foot injuries living in two provinces of Iran. An information form for demographic data and injury-related factors was used. Additionally, the previously validated Persian version of the Symptom Checklist-90-Revision (SCL-90-R) questionnaire was used for data collection.

**Results:**

The respondents were 215 male veterans with a mean age of 51.7 ± 7.5 years. The most common mental health problems were observed for the somatization (24.7%), obsessions-compulsions (14.4%), and anxiety (12.6%). Based on the Global Severity Index (GSI), 48.6% of individuals had a possible psychiatric/psychological illness. According to the multivariate regression analysis, GSI scores were significantly higher among veterans who were older than 27 years at the time of injury (*P* = 0.005), had an associated injury (*P* = 0.002), and had a history of hospitalization within the past 12 months for reasons other than their injury (*P* = 0.035).

**Conclusions:**

Approximately half of the combat veterans with ankle-foot neuromusculoskeletal disorders likely had psychological problems. The evaluation of the patterns and predicting factors of psychological conditions may inform strategic planning efforts and decision-making, which, in turn, may provide a better quality of life for veterans. Further studies that utilize longitudinal designs are needed to evaluate and compare the psychological status of different groups of veterans and other groups in the general population.

## Background

Approximately 70% of all battlefield damages are musculoskeletal injuries, which are associated with a high morbidity despite its low mortality rate [1, 2]. About two-thirds of all war injuries in both survivors and victims are related to upper and lower extremities, and more than half are open-wound injuries. However, combat-related craniofacial, cervical, thoracic, and abdominal injuries are mostly linked with a high rate of mortality [3]. The injuries of extremities, along with limb amputation, can cause neuromusculoskeletal disorders, all of which may lead to disability, reduced quality of life, and consequently, poorer mental health among war survivors [4].

After nearly three decades of the Iran-Iraq war, there are more than 500,000 Iranian veterans registered with the Veterans and Martyrs Affairs Foundation (VMAF) [5]. After the end of the Iran-Iraq war, several researchers investigated the health-related issues among Iranian war veterans with physical and mental health disorders. Their researches on these issues span a variety of topics, few of which focus on mental health problems among veterans and their families [6–9].

Studies in different countries have found high rates of mental disorders among veterans, especially posttraumatic stress disorder (PTSD) and depression [10]. There are several studies that reported a high prevalence of mental disorders such as depression, psychosis, anxiety, paranoia, and hypochondriasis among surviving veterans, from those serving in World War II to the Persian Gulf War [11]. Similar results have been described in Iranian studies, with some reporting a high Global Severity Index (GSI) above the cutoff point of the Symptom Checklist-90-Revised (SCL-90-R) in more than 95% of Iranian veterans sustaining physical and chemical injury [12], as well as high rates of depression, anxiety, anger, and aggression [13]. However, veterans with purely physical disorders, such as ankle-foot neuromusculoskeletal disorders, are often neglected in psychological assessments because mental health evaluations are usually focused on those with a psychological disturbance or with a high percentage of injury. Moreover, with the use of orthotics, especially ankle-foot orthoses and prostheses, these parts of the body are usually covered by clothes. Thus, this appearance may mask the depth of the influence of the physical problem on the individual’s psychological well-being, and as a result, veterans with such injuries may be assumed to have normal mental health despite their disability. According to the VMAF, 10,227 ankle-foot injured veterans have survived the Iran-Iraq war, and most of them have suffered two or more injuries. The hypothesis of this study was that having ankle-foot neuromusculoskeletal injuries would be associated with high rates of psychological problems among veterans. Thus, this study was designed to evaluate the psychological status of combat veterans with ankle-foot neuromusculoskeletal problems.

## Methods

We conducted a cross-sectional, descriptive survey between 2014 and 2016. The statistical population consisted of veterans with ankle-foot neuromusculoskeletal disorders registered in the local branch of the VMAF in two provinces of Iran including Zanjan and Markazi [14]. A telephone call invitation was made to all veterans asking them to participate in the study. Of 393 eligible veterans with ankle-foot injuries, 215 accepted to participate in the study and were enrolled through census sampling.

The study protocol was approved by the ethics committees of Janbazan Medical and Engineering Research Center (JMERC) and performed in accordance with the Declaration of Helsinki and its subsequent revisions. All participants were informed about the voluntary nature of their participation and the aims of the survey. Verbal informed consent was obtained from each participant.

The first part of data collection was accomplished using an information form developed for the study that assessed demographic data such as age, gender, marital status (and number of children), education level, occupation, economic level, percentage of disability, and history of hospitalization within the past 12 months. These data were obtained through 10 min-interviews with the participants.

The second part of data collection assessed information on mental health status, obtained using the Persian version of SCL-90-R, which is a multi-dimensional self-reported symptom inventory designed to measure current psychological symptom status. This assessment requires approximately 12–20 min to administer. It consists of 90 items on nine primary symptom dimensions, including somatization, obsessions-compulsions, interpersonal sensitivity, depression, anxiety, hostility, phobic anxiety, paranoid ideation, and psychoticism. Each of the dimensions is assessed by 6–13 items scored on a Likert-type scale, with response options for each item ranging from zero (“never”) to four (“very often”). The overall score for each dimension is the mean score of all items on the subscale and directly reflects the severity of the mental health problem. According to previous studies, subscale scores ≥2 were suggestive of potential mental health issues [15, 16]. The reliability and validity of this questionnaire have been demonstrated by several studies [17]. The mean reliability of each factor was estimated to have a Cronbach’s alpha of 0.97. This study used the GSI, which is the average score of the 90 items and indicates the current level or depth of a given disorder [18]. Based on previous studies, a cut-off value of 1.3 was used for the GSI [15]. Given the self-report format, ease of use, and previous reliability and validity of the Persian version of the SCL-90-R in Iran, this instrument was used for the initial mental health screening evaluation in our population. In the last step, all veterans who were suspected of having mental health problems were referred for a visit with a psychiatrist.

Quantitative data with normal distributions are presented as the means ± standard deviations (SDs), and categorical variables are shown as frequencies and proportions. A two-tailed Student’s t-test or ANOVA was used after establishing the normal distribution of data and homogeneity of variances for continuous values. Post hoc analysis was used to compare the means across the study groups. A multiple linear stepwise regression analysis was performed to evaluate the independent contribution of demographic characteristics and injury-related factors to GSI scores. A two-tailed alpha with *P* < 0.05 was considered significant. The analyses were conducted using SPSS software, version 20.0 (SPSS Inc., Chicago, IL, USA).

## Results

In total, 215 veterans participated and provided an acceptable and reliable response to the questionnaire; thus the response rate was 54.7% in our study. Since all Iranian participants in the battlefields of the Iran-Iraq war were male, all veterans were men with a mean age of 51.7 ± 7.5 (range: 29–92) years. The average percentage of injury (disability rating) was 32.8 ± 13.7% (range: 5%–70%) among participating veterans. The mean age at the time of injury was 27.9 ± 3.3 (range: 15–35) years. In total, 156 (72.6%) had an associated injury other than ankle-foot neuromusculoskeletal problems. The associated injuries included psychological problems, chemical injuries, and spinal cord damage in 49 (22.8%), 19 (8.8%), and 6 (2.8%) individuals, respectively. Additionally, other associated physical injuries (e.g., head, facial, thoracic, abdominal, upper limb, back and spinal, or other injuries) were seen in 125 (58.6%) veterans. More detailed demographic characteristics of our study population have been published elsewhere [14].

Tables 1 and 2 show demographic characteristics and injury-related variables, as well as their associations with SCL-90-R scores. The GSI was significantly associated with higher scores for “having more than four children” (*P* = 0.045; Table 1). As seen in Table 1, all demographic variables (except age), as well as most of the injury-related variables, were significantly associated with at least one of the SCL-90-R subscale scores. Education level was significantly linked with the subscales for obsessions-compulsions (*P* = 0.038), interpersonal sensitivity (*P* = 0.043), and depression (*P* = 0.045), with higher scores seen among participants in the “less than diploma” group. Veterans who had more than four children had a higher degree of somatization (*P* = 0.041), interpersonal sensitivity (*P* = 0.027), depression (P = 0.041), and anxiety (*P* = 0.008). Post hoc analysis indicated a significant increase in the somatization subscale among retired individuals compared with those who were government employees (*P* = 0.013).

**Table 1:** Association of demographic characteristics with psychological symptoms in the SCL-90-R (*n* = 215)

Item	*n*(%)	SCL-90-R subscales									GSI
		Somatization	Obsessions-compulsions	Interpersonal sensitivity	Depression	Anxiety	Hostility	Phobic anxiety	Paranoid ideation	Psychoticism	
Age(year)											
≤ 40	5(2.3%)	1.8 ± 0.3	1.5 ± 0.2	1.6 ± 0.3	1.4 ± 0.3	1.3 ± 0.4	1.5 ± 0.4	1.3 ± 0.7	0.9 ± 0.4	1.2 ± 0.7	1.4 ± 0.2
41–60	191(88.8%)	1.5 ± 0.7	1.3 ± 0.7	1.1 ± 0.7	1.2 ± 0.7	1.2 ± 0.7	1.2 ± 0.7	0.8 ± 0.7	1.0 ± 0.8	0.9 ± 0.7	1.2 ± 0.6
> 60	19(8.8%)	1.5 ± 0.9	1.3 ± 0.7	1.2 ± 0.7	1.3 ± 0.7	1.2 ± 0.8	1.1 ± 0.7	0.8 ± 0.7	1.0 ± 0.7	0.9 ± 0.7	1.2 ± 0.7
*P* value		0.609	0.834	0.287	0.814	0.961	0.620	0.356	0.907	0.718	0.683
Education											
Less than diploma	127(59.1%)	1.5 ± 0.7	1.4 ± 0.7	1.2 ± 0.7	1.3 ± 0.7	1.3 ± 0.7	1.3 ± 0.7	0.9 ± 0.7	1.1 ± 0.8	1.0 ± 0.7	1.3 ± 0.6
Diploma	69(32.1%)	1.5 ± 0.7	1.2 ± 0.7	1.0 ± 0.7	1.1 ± 0.7	1.2 ± 0.7	1.1 ± 0.7	0.7 ± 0.6	1.0 ± 0.8	0.8 ± 0.7	1.1 ± 0.6
Higher education	19(8.8%)	1.3 ± 0.8	1.1 ± 0.7	0.9 ± 0.6	1.1 ± 0.7	1.0 ± 0.7	0.9 ± 0.7	0.9 ± 0.8	0.9 ± 0.9	0.8 ± 0.8	1.0 ± 0.6
P value		0.408	0.038	0.043	0.045	0.204	0.065	0.078	0.359	0.214	0.066
Number of children in the family											
1–2	65(30.2%)	1.4 ± 0.6	1.2 ± 0.7	1.0 ± 0.6	1.1 ± 0.7	1.1 ± 0.7	1.2 ± 0.7	0.8 ± 0.7	1.0 ± 0.8	0.8 ± 0.7	1.1 ± 0.6
3–4	99(46.0%)	1.4 ± 0.8	1.3 ± 0.7	1.2 ± 0.7	1.2 ± 0.7	1.2 ± 0.7	1.2 ± 0.8	0.8 ± 0.6	1.1 ± 0.8	0.9 ± 0.7	1.2 ± 0.6
> 4	51(23.7%)	1.7 ± 0.7	1.4 ± 0.6	1.3 ± 0.7	1.4 ± 0.6	1.5 ± 0.6	1.3 ± 0.6	1.0 ± 0.8	1.1 ± 0.7	1.1 ± 0.7	1.4 ± 0.5
P value		0.041	0.177	0.027	0.041	0.008	0.647	0.080	0.833	0.087	0.045
Job status											
Government employee	42(19.5%)	1.3 ± 0.7	1.1 ± 0.8	1.0 ± 0.7	1.0 ± 0.8	1.0 ± 0.8	1.1 ± 0.8	0.7 ± 0.7	1.0 ± 0.9	0.8 ± 0.7	1.0 ± 0.7
Self-employed	28(13.0%)	1.4 ± 0.7	1.3 ± 0.6	1.2 ± 0.6	1.1 ± 0.6	1.1 ± 0.6	1.1 ± 0.6	0.8 ± 0.7	1.0 ± 0.6	0.9 ± 0.7	1.1 ± 0.5
Retired	50(23.3%)	1.7 ± 0.7	1.4 ± 0.6	1.2 ± 0.5	1.3 ± 0.6	1.4 ± 0.6	1.2 ± 0.5	0.9 ± 0.5	1.1 ± 0.7	1.0 ± 0.6	1.3 ± 0.5
Unemployed	95(44.2%)	1.5 ± 0.7	1.3 ± 0.7	1.2 ± 0.7	1.3 ± 0.7	1.3 ± 0.7	1.3 ± 0.8	0.9 ± 0.7	1.1 ± 0.8	1.0 ± 0.7	1.2 ± 0.6
P value		0.013	0.299	0.220	0.056	0.058	0.607	0.575	0.868	0.606	0.137

**Table 2:** Association of injury-related factors with psychological symptoms in the SCL-90-R (*n* = 215)

Item	n (%)	SCL-90-R subscales									GSI
		Somatization	Obsessions-compulsions	Interpersonal sensitivity	Depression	Anxiety	Hostility	Phobic anxiety	Paranoid ideation	Psychoticism	
Disability rating											
< 30%	94(43.7%)	1.5 ± 0.6	1.4 ± 0.7	1.3 ± 0.7	1.3 ± 0.6	1.3 ± 0.7	1.3 ± 0.7	1.0 ± 0.7	1.2 ± 0.7	1.0 ± 0.7	1.3 ± 0.6
≥ 30%	121(56.3%)	1.5 ± 0.8	1.2 ± 0.7	1.1 ± 0.7	1.1 ± 0.7	1.2 ± 0.7	1.1 ± 0.8	0.7 ± 0.6	1.0 ± 0.8	0.9 ± 0.7	1.1 ± 0.6
*P* value		0.921	0.018	0.027	0.144	0.103	0.186	0.005	0.064	0.132	0.062
Age at the time of injury (year)											
≤ 27	105(48.8%)	1.4 ± 0.7	1.2 ± 0.8	1.0 ± 0.7	1.0 ± 0.7	1.1 ± 0.8	1.1 ± 0.8	0.8 ± 0.8	0.9 ± 0.8	0.8 ± 0.7	1.1 ± 0.6
> 27	110(51.2%)	1.6 ± 0.7	1.4 ± 0.6	1.3 ± 0.6	1.3 ± 0.7	1.3 ± 0.6	1.3 ± 0.6	0.9 ± 0.6	1.2 ± 0.8	1.0 ± 0.7	1.3 ± 0.6
*P* value		0.093	0.069	0.007	0.002	0.043	0.211	0.528	0.006	0.069	0.017
Associate injury											
No	59(27.4%)	1.3 ± 0.8	1.2 ± 0.8	1.0 ± 0.7	1.1 ± 0.7	1.0 ± 0.7	1.0 ± 0.7	0.7 ± 0.7	0.9 ± 0.8	0.8 ± 0.7	1.0 ± 0.6
Yes	156(72.6%)	1.6 ± 0.7	1.3 ± 0.7	1.2 ± 0.6	1.2 ± 0.7	1.3 ± 0.7	1.3 ± 0.7	0.9 ± 0.7	1.1 ± 0.8	1.0 ± 0.7	1.2 ± 0.6
*P* value		0.006	0.106	0.162	0.083	0.007	0.003	0.110	0.159	0.104	0.022
Limb disability											
No	72(33.5%)	1.2 ± 0.8	1.1 ± 0.8	1.0 ± 0.7	1.0 ± 0.7	0.9 ± 0.7	0.9 ± 0.7	0.6 ± 0.7	0.8 ± 0.7	0.7 ± 0.7	0.9 ± 0.6
Yes	143(66.5%)	1.7 ± 0.6	1.4 ± 0.6	1.2 ± 0.6	1.3 ± 0.7	1.4 ± 0.6	1.3 ± 0.7	1.0 ± 0.7	1.2 ± 0.8	1.0 ± 0.7	1.3 ± 0.6
*P* value		<0.001	<0.001	0.005	0.001	<0.001	<0.001	0.001	0.001	0.001	<0.001
Head injury											
No	169(78.6%)	1.4 ± 0.6	1.5 ± 0.7	1.4 ± 0.6	1.4 ± 0.7	1.4 ± 0.6	1.3 ± 0.6	1.2 ± 0.7	1.4 ± 0.7	1.2 ± 0.6	1.4 ± 0.5
Yes	46(21.4%)	1.5 ± 0.7	1.3 ± 0.7	1.1 ± 0.7	1.1 ± 0.7	1.2 ± 0.7	1.2 ± 0.7	0.8 ± 0.7	0.9 ± 0.8	0.8 ± 0.7	1.1 ± 0.6
*P* value		0.004	0.161	0.126	0.199	0.014	0.128	0.202	0.432	0.061	0.039
Facial injury											
No	177(82.3%)	1.4 ± 0.7	1.3 ± 0.7	1.1 ± 0.7	1.2 ± 0.7	1.2 ± 0.7	1.1 ± 0.7	0.8 ± 0.7	1.0 ± 0.8	0.9 ± 0.7	1.1 ± 0.6
Yes	38(17.7%)	1.9 ± 0.6	1.4 ± 0.5	1.2 ± 0.6	1.3 ± 0.6	1.5 ± 0.5	1.5 ± 0.7	0.9 ± 0.6	1.1 ± 0.7	1.0 ± 0.6	1.4 ± 0.5
*P* value		0.001	0.230	0.310	0.138	0.008	0.011	0.545	0.484	0.239	0.050
Thoracic injury											
No	191(88.8%)	1.5 ± 0.7	1.3 ± 0.7	1.1 ± 0.7	1.2 ± 0.7	1.2 ± 0.7	1.2 ± 0.7	0.8 ± 0.7	1.0 ± 0.8	0.9 ± 0.7	1.2 ± 0.6
Yes	24(11.2%)	1.8 ± 0.7	1.5 ± 0.6	1.3 ± 0.7	1.4 ± 0.8	1.4 ± 0.7	1.3 ± 0.7	1.0 ± 0.7	1.1 ± 0.7	1.1 ± 0.8	1.4 ± 0.6
*P* value		0.056	0.129	0.252	0.238	0.123	0.28	0.303	0.845	0.120	0.133
Abdominal injury											
No	171(79.5%)	1.4 ± 0.7	1.3 ± 0.7	1.1 ± 0.7	1.2 ± 0.7	1.2 ± 0.7	1.2 ± 0.7	0.8 ± 0.7	1.0 ± 0.8	0.9 ± 0.7	1.1 ± 0.6
Yes	44(20.5%)	1.8 ± 0.6	1.5 ± 0.7	1.3 ± 0.7	1.3 ± 0.7	1.4 ± 0.7	1.3 ± 0.7	1.0 ± 0.6	1.2 ± 0.8	1.1 ± 0.7	1.4 ± 0.6
*P* value		0.004	0.111	0.091	0.322	0.027	0.138	0.194	0.097	0.021	0.033
Upper limb injury											
No	155(72.1%)	1.4 ± 0.7	1.3 ± 0.7	1.2 ± 0.7	1.2 ± 0.7	1.2 ± 0.7	1.2 ± 0.7	0.9 ± 0.7	1.1 ± 0.8	0.9 ± 0.7	1.2 ± 0.6
Yes	60(27.9%)	1.6 ± 0.6	1.4 ± 0.6	1.1 ± 0.6	1.2 ± 0.6	1.3 ± 0.6	1.3 ± 0.7	0.9 ± 0.7	0.9 ± 0.7	0.8 ± 0.6	1.2 ± 0.5
*P* value		0.077	0.488	0.585	0.911	0.367	0.206	0.790	0.049	0.321	0.852
Back and spinal injury											
No	178(82.8%)	1.5 ± 0.7	1.3 ± 0.7	1.1 ± 0.7	1.2 ± 0.7	1.2 ± 0.7	1.2 ± 0.7	0.9 ± 0.7	1.1 ± 0.8	0.9 ± 0.7	1.2 ± 0.6
Yes	37(17.2%)	1.6 ± 0.8	1.3 ± 0.6	1.2 ± 0.7	1.2 ± 0.6	1.3 ± 0.7	1.3 ± 0.7	0.9 ± 0.7	1.0 ± 0.8	1.0 ± 0.7	1.2 ± 0.6
*P* value		0.191	0.797	0.479	0.907	0.691	0.192	0.984	0.801	0.530	0.566
Hospitalization due to injury											
No	184(85.6%)	1.4 ± 0.7	1.3 ± 0.7	1.1 ± 0.7	1.1 ± 0.7	1.2 ± 0.7	1.2 ± 0.7	0.8 ± 0.7	1.0 ± 0.7	0.9 ± 0.7	1.1 ± 0.6
Yes	31(14.4%)	1.8 ± 0.7	1.5 ± 0.7	1.3 ± 0.7	1.5 ± 0.8	1.5 ± 0.7	1.4 ± 0.7	0.9 ± 0.7	1.3 ± 0.9	1.2 ± 0.7	1.4 ± 0.6
*P* value		0.009	0.052	0.104	0.010	0.027	0.057	0.478	0.035	0.026	0.016
Hospitalization for other reasons											
No	190(88.4%)	1.5 ± 0.7	1.3 ± 0.7	1.1 ± 0.7	1.2 ± 0.7	1.2 ± 0.7	1.2 ± 0.7	0.9 ± 0.7	1.0 ± 0.8	0.9 ± 0.7	1.2 ± 0.6
Yes	25(11.6%)	1.9 ± 0.6	1.6 ± 0.6	1.4 ± 0.5	1.5 ± 0.6	1.4 ± 0.6	1.3 ± 0.6	0.9 ± 0.6	1.3 ± 0.7	1.0 ± 0.6	1.4 ± 0.4
*P* value		0.003	0.044	0.050	0.013	0.132	0.366	0.662	0.120	0.733	0.039

There were also some factors associated with higher GSI scores among injury-related variables, including “age at the time of injury >27 years” (*P* = 0.017), “presence of an associated injury” (*P* = 0.022), “presence of abdominal injury” (*P* = 0.033), “presence of a limb disability” (*P* < 0.001), “hospitalization for the reason of injury” (*P* = 0.016), and “hospitalization for other reasons” (*P* = 0.039). Additionally, head injury was significantly associated with lower GSI scores (*P* = 0.039). Among the damage-related factors, disability percentage of less than 30% was significantly associated with higher scores on the subscales for obsessions-compulsions (*P* = 0.018), interpersonal sensitivity (*P* = 0.027), and phobic anxiety (*P* = 0.005). Age at the time of injury >27 years was related to higher scores on the subscales for interpersonal sensitivity (*P* = 0.007), depression (*P* = 0.002), anxiety (*P* = 0.043), and paranoid ideation (*P* = 0.006). All subscale scores were significantly higher in the presence of limb disability (*P* < 0.05). Having an associated injury in addition to ankle-foot neuromusculoskeletal problems was related to higher somatization (*P* = 0.006), anxiety (*P* = 0.007), and hostility (*P* = 0.003) scores. Injuries to different parts of the body were also associated with either higher or lower scores on the psychological subscales (Table 2). Additionally, hospitalization due to injury or other reasons was significantly associated with higher scores on all psychological subscales (except hostility and phobic anxiety).

The mean GSI score was 1.2 ± 0.6, and the mean SCL-90-R scores for somatization, obsessions-compulsions, interpersonal sensitivity, depression, anxiety, hostility, phobic anxiety, paranoid ideation, and psychoticism subscales were 1.5 ± 0.7, 1.3 ± 0.7, 1.1 ± 0.7, 1.2 ± 0.7, 1.2 ± 0.7, 1.2 ± 0.7, 0.9 ± 0.7, 1.0 ± 0.8, and 0.9 ± 0.7, respectively. Based on the GSI cut-off point of 1.3, there were a total of 105 (48.6%) individuals with possible psychiatric/psychological illness within the study sample. Table 3 shows the frequency of psychological problems based on cut-off points for the SCL-90-R subscales. The highest scores were seen in the somatization and obsessions-compulsions subscales, and as seen in Table 3, the most common psychological problems were related to the somatization, obsessions-compulsions, and anxiety subscales, with rates of 24.7%, 14.4%, and 12.6%, respectively.

**Table 3:** The categorized SCL-90-R subscale scores of veterans with ankle-foot neuromusculoskeletal problems (*n* = 215)

Item	Normal (<1)	Suspected (1–2)	Psychiatric/psychological illness (>2)
Somatization	48 (22.3%)	114 (53.0%)	53 (24.7%)
Obsessions-compulsions	65 (30.2%)	119 (55.3%)	31 (14.4%)
Interpersonal sensitivity	77 (35.8%)	122 (56.7%)	16 (7.5%)
Depression	82 (38.1%)	113 (52.6%)	20 (9.3%)
Anxiety	78 (36.3%)	110 (51.2%)	27 (12.6%)
Hostility	78 (36.3%)	115 (53.5%)	22 (10.2%)
Phobic anxiety	120 (55.8%)	86 (40.0%)	9 (4.2%)
Paranoid ideation	96 (44.6%)	101 (47.0%)	18 (8.4%)
Psychoticism	112 (52.1%)	93 (43.3%)	10 (4.6%)

A multivariate regression analysis showed that significant injury-related factors for higher GSI scores included “age at the time of injury” (*P* = 0.005), “presence of an associated injury” (*P* = 0.002), and “hospitalization due to reasons other than the injury” (*P* = 0.035).

## Discussion

Our results indicated that the most prevalent mental health problems were somatization, obsessions-compulsions, and anxiety. In a study on Iraq/Afghanistan war veterans diagnosed with PTSD, Kimbrel et al. [19] showed similarly high proportions for these mental disorders in their sample, although their sample showed higher rates. Many studies have reported an association between exposure to trauma, especially in childhood, and psychological disorders such as hypochondriasis [20], somatization [21, 22], obsessive-compulsive disorder (OCD) [23–25], and anxiety [26], as well as other mental health problems [19]. Moreover, Kimbrel et al. [19] stated that psychological conditions such as somatization, OCD, hypochondriasis, and alcohol use disorder were also prominently elevated among veterans without PTSD, suggesting that the occurrence of these psychological disorders was not simply due to their association with PTSD. Other Iranian studies on veterans have reported results similar to those in our study. Research on combat veterans has indicated that somatization and hypochondriasis are the most common psychological disorders [11]. In a study by Zargar et al. [27] on 330 veterans in Isfahan, Iran, it was found that the greatest variance between the SCL-90-R subscales was related to somatization and anxiety. These findings may pose new considerations for the development of strategies to diagnose and treat mental health problems among all veterans suffering physical and chemical injury, in addition to those with PTSD and other psychological disorders.

Our study also presented some factors associated with mental health status in veterans. The main factors linked with greater GSI scores, which indicated worse mental health, were age at the time of injury, presence of additional injuries, number of family members, hospitalization, and education.

Our findings showed a higher severity of mental health problems for veterans whose injuries had occurred at age 27 or older at the time of injury. It has been shown that the experience of traumatic events at older ages, including late adolescence or early adulthood, is linked with several problems in interpersonal, social, and occupational functioning [27]. Furthermore, soldiers who were older at the time of injury may have had more exposure to traumatic events in combat, may have moved to higher command positions that placed more responsibility on them, and may have had more stable familial, social, occupational, and economic situations, all of which may have led to impaired mental health due to injury-related outcomes.

In general, mental disorders may be consequences of occupational functioning, such that negative job-related situations (such as job dissatisfaction or unemployment) among veterans may lead to depression [27, 28]. Moreover, it has been shown that one of the most important factors influencing the mental health of veterans is lack of an appropriate occupation [29], and while they are employed, the risk of depression and other mental health disorders may decrease [30]. In the absence of a job, veterans lose employment-related supports, not only those associated with economic status, and the resulting effects on physical, cognitive, psychological, and social functioning can all lead to psychological stress, especially when they had a job before their injury [31]. The damage in occupational functioning may lead to economic problems, which were also significantly associated with psychological problems in our study. Other variables linked with veterans’ economic situations, such as number of family members, were also associated with higher scores on the SCL-90-R subscales and the GSI. Taken together, these findings emphasize the influence of economic status on mental health in veterans.

Additionally, veterans with associated injuries (other than ankle-foot problems) represented a large category, with several factors related to the severity of psychological distress. Although psychological problems (e.g., PTSD) were a prevalent associated injury in our study population and may directly influence SCL-90-R scores or lead to hospitalizations, veterans with chemical injuries and other associated physical damages should receive more attention in mental health services, and interventions for prevention or treatment of affected veterans should be implemented.

One of the limitations of this study was its cross-sectional design, which should be enhanced by following these veterans in a longitudinal study to evaluate changes in their mental health over time and the effects of interventions (i.e., treatment and rehabilitation). Another limitation of our study was the response rate of 54.7%. While this rate may be excellent for other descriptive studies, our research evaluated mental health status. Thus, the current response rate may lead to an under estimation of prevalence by masking the veterans whose illness and its severity (physical or psychological) did not permit them to participate in the survey. The third limitation was that the study included veterans in only two provinces of Iran. Veterans living in the other 29 provinces, especially in Iran-Iraq border provinces, who may have more severe problems due to their proximity to conflict zones and suffer from injuries in events such as land mine accidents, were not included in the study.

## Conclusions

Combat veterans with ankle-foot neuromusculoskeletal problems may be a neglected group in mental health assessments. Psychological problems may lead to a lower quality of life in this population. However, evaluation of the patterns and predicting factors of these psychological problems may support the health services and other relevant entities in strategic planning and decision-making, which may eventually provide a better quality of life for them. It is recommended that veterans with higher scores on the SCL-90-R subscales undergo complementary assessments and current diagnostic systems for mental health, such as the Diagnostic and Statistical Manual of Mental Disorders (DSM-V); using this approach, we can better detect the veterans who may need treatment or intervention. Furthermore, mental health screening using the SCL-90-R can be recommended as part of the routine evaluation of all medical and health-related affairs involving veterans to better predict and detect potential psychological problems in this population and to implement strategies to prevent the development of psychological disorders. Further studies that utilize longitudinal designs are needed to evaluate and compare the psychological status of different groups of veterans as well as groups in the general population.

## Abbreviations

CI: Confidence Interval
GSI: Global Severity Index
JMERC: Janbazan Medical and Engineering Research Center
PTSD: Posttraumatic Stress Disorder
SCL-90-R: Symptom Checklist-90-Revision
VMAF: Veterans and Martyrs Affairs Foundation
